# Definition of the symbiovar viciae in the species *Rhizobium azibense* and biogeographic implications

**DOI:** 10.1007/s00203-022-03330-w

**Published:** 2022-12-08

**Authors:** Abdelaal Shamseldin, Alvaro Peix, Encarna Velázquez

**Affiliations:** 1 Environmental Biotechnology Department, Genetic Engineering and Biotechnology Research Institute (GEBRI), City of Scientific Research and Technology Applications (SRTA-City), New Borg El-Arab, Alexandria Egypt; 2 Instituto de Recursos Naturales y Agrobiología, IRNASA-CSIC, Salamanca, Spain; 3 Grupo de Interacción Planta-Microorganismo, USAL, Unidad Asociada al CSIC por el IRNASA, Salamanca, Spain; 4 Departamento de Microbiología y Genética, Universidad de Salamanca, Salamanca, Spain; 5 Instituto de Investigación en Agrobiotecnología (CIALE), Universidad de Salamanca, Salamanca, Spain

**Keywords:** *Rhizobium azibense*, Symbiovar viciae, *Vicia faba*, Egypt

## Abstract

*Vicia faba* L. (faba bean) is a legume cultivated worldwide which commonly establishes effective symbiosis with the symbiovar viciae of species from the *Rhizobium leguminosarum* phylogenetic group. However, on the basis of the *rrs, recA,* and *atpD* gene phylogenies, in this work we identified a strain named EFBRI 42 nodulating *V. faba* as *Rhizobium azibense*. This is the first report on the nodulation of *Vicia* by *R. azibense* which commonly nodulates *P. vulgaris* and to date encompasses strains harboring the *nodC* genes typical of the symbiovars gallicum and phaseoli. However, the strain EFBRI 42 carries a *nodC* gene typical of the symbiovar viciae for which we report here by the first time this symbiovar in *R. azibense.* This finding showed the existence of symbiotic genes horizontal transfer events during the coevolution of *R. azibense* with *P. vulgaris* and *V. faba* in their respective distribution centers of Mesoamerica and the Middle East.

*Vicia faba* L. (faba bean) is a legume probably indigenous to the Near East (Cubero 1974) which is currently cultivated worldwide for the high nutritive value of its seeds, its usefulness as forage and cover crop, and by its ability for nitrogen fixation in symbiosis with rhizobia (Etemadi et al. 2019; Maaluf et al. 2019).

As other legumes, *V. faba* establishes nitrogen-fixing symbiosis with rhizobial strains whose diversity has been studied in several countries of North Africa where this legume is mostly nodulated by strains phylogenetically related to *Rhizobium leguminosarum*, *R. laguerreae* and *R. etli* (Shamseldin et al. 2009; Youseif et al. 2014; Hassan et al. 2015; Belhadi et al. 2018; Benidire et al. 2018; Missbah El Idrissi et al. 2020).

Several of these studies have been carried out in Egypt where this legume has been cultivated for many centuries (Shamseldin et al., 2009; Youseif et al. 2014; Hassan et al. 2015) showing that the strains nodulating *V. faba* mostly belong to the symbiovar viciae of *Rhizobium leguminosarum* and *R. etli* (Shamseldin et al. 2009; Youseif et al. 2014; Hassan et al. 2015); which is a symbiotic variant able to nodulate specifically legumes of the *Vicia* cross inoculation group (Rogel et al. 2011; Peix et al. 2015). However, some strains effectively nodulating faba bean have not been assigned to a species and symbiovar until now, as occurs with the strain EFBRI 42 isolated in Egypt (Shamseldin et al., 2009). Therefore, in the present study, we identified this strain through the analysis of the core genes, like *rrs*, *recA,* and *atpD*, and the symbiotic gene *nodC*, which were not previously analyzed for this strain (Shamseldin et al. 2009), and are commonly used for the rhizobia identification at species and symbiovar levels (Peix et al. 2015).

To obtain sequences of these genes, we extracted DNA of the strain EFBRI 42 grown on TY plates (Triptone Yeast Agar) [(Beringer)] during 24 h at 28 °C. Genomic DNA was obtained using the DNeasy UltraClean Microbial DNA Isolation Kit (Qiagen) following the manufacturer’s protocol. The amplification and sequencing of *rrs*, *recA*, *atpD* and *nodC* genes were carried out in the conditions and with the primers previously reported (Carro et al. 2012; Gaunt et al. 2001; Laguerre et al. 2001).

The obtained sequences were compared with those from GenBank using the BLASTN program (Altschul et al. 1990) and the sequences of the closely related bacteria were downloaded from GenBank for phylogenetic analyses. The sequences were aligned using the Clustal W program (Thompson et al. 1977). The phylogenetic distances were calculated according to Kimura’s two-parameter model (Kimura 1980). The phylogenetic trees were inferred using the neighbor joining model (Saitou and Nei 1987) MEGA 7.0 (Kumar et al. 2016) was used for all phylogenetic analyses (Figs. 1 and 2).

**Fig. 1:**
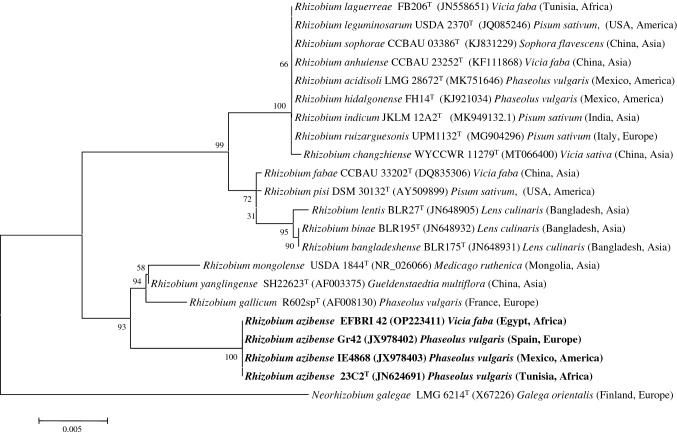
Neighbor-joining phylogenetic tree based on *rrs* gene sequences (1310 nt) showing the taxonomic location of the strain EFBRI 42 within the genus *Rhizobium*. Bootstrap values calculated for 1000 replications are indicated. Bar: 5 nt substitution per 1000 nt. Accession numbers from Genbank are given in brackets

**Fig. 2:**
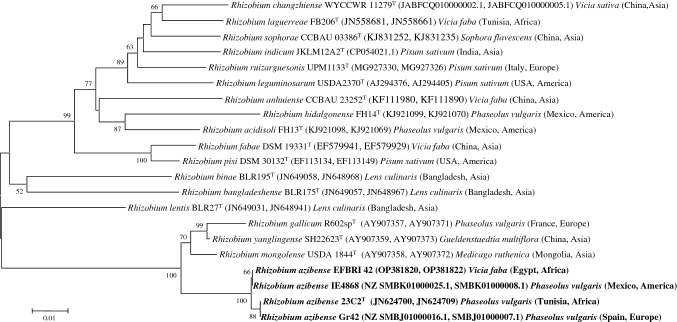
Neighbor-joining phylogenetic tree based on *recA* and *atpD* concatenated gene sequences (700 nt) showing the position of the strain EFBRI 42 within the genus *Rhizobium*. Bootstrap values calculated for 1000 replications are indicated. Bar, 1 nt substitution per 100 nt. Accession numbers from Genbank are given in brackets

The species from the phylogenetic group of *R. leguminosarum* are the most common endosymbionts of *Vicia* species and other legumes from its cross inoculation group as *Pisum* and *Lens* (Fig. 1). However, the strain EFBRI 42, isolated in Egypt, represented a separate genotype from *R. leguminosarum* according to the *rrs-*RFLP pattern analysis (Shamseldin et al. 2009). Accordingly, the sequence of *rrs* gene of strain EFBRI 42 showed 100% similarity with respect to the species *Rhizobium azibense*, which encompasses strains nodulating *P. vulgaris* in different continents and belongs to a group phylogenetically divergent of *R. leguminosarum* (Fig. 1).

The four species of this phylogenetic group, *R. azibense*, *R. gallicum*, *R. mongolense,* and *R. yanglingense*, have closely related *rrs* genes and therefore, the identification of the strain EFBRI 42 was confirmed by the analysis of the *recA* and *atpD* housekeeping genes, which allowed the differentiation of *Rhizobium* species with closely related *rrs* genes (Peix et al. 2015). The results of the concatenated *recA* and *atpD* gene sequences confirmed the affiliation of the strain EFBRI 42 to *R. azibense* with similarity values higher than 99.0% in both genes (Fig. 2). These results constitute the first report on the nodulation of *V. faba* by *R. azibense*, which to date only included strains isolated from *P. vulgaris* nodules (Mnasri et al. 2014).

The identification at symbiovar level is mainly based on the *nodC* gene analysis in the case of the genus *Rhizobium* (Peix et al. 2015). Based on the results of this analysis the Egyptian strain EFBRI 42 belongs to the symbiovar viciae with its *nodC* gene being closely related (higher than 98% similarity) to *Rhizobium* strains isolated from *Lens culinaris* nodules in Morocco, Syria and Iran (Fig. 3). This is the first report of the symbiovar viciae within the species *R. azibense* which to date only contains strains nodulating *P. vulgaris* belonging to the symbiovars gallicum and phaseoli (Fig. 3). The strains of the species *R. azibense* have been isolated in three different continents, Africa, America and Europe (Mnasri et al. 2014). The strains 23C2^T^ isolated in Tunisia (Africa) and IE4868 isolated in Mexico (America) belong to the symbiovar gallicum, whereas the strain GR42 isolated in Spain (Europe) belongs to the symbiovar phaseoli (Fig. 3). Both symbiovars, phaseoli and gallicum, have been isolated from *P. vulgaris* nodules in its distribution centers (Silva et al. 2003; Mnasri et al. 2014; Bustos et al. 2017), which are located in Mesoamerica (Bitocchi et al. 2012). Therefore, probably the symbiotic genes typical of these symbiovars arrived to Europe and Africa together with the seeds of *P. vulgaris* and were transferred to strains of species indigenous to these continents. This seems to be clear in the case of *R. leguminosarum* strains carrying the symbiovar phaseoli, because an origin outside America has been proposed for this species (Alvarez-Martínez et al. 2009), but still there are not enough data to hypothesize on the geographical origin of the species *R. azibense*. Nevertheless, the fact of the existence of the symbiovar viciae within this species opens the door to think that this species has coevolved for long times with Vicia species whose geographical origin has been located in a region of the Middle East that include Egypt (Caracuta et al. 2015). In any case, the existence within *R. azibense* of three symbiovars to date nodulating legumes indigenous to different continents, as occurs with *P. vulgaris* and *V. faba*, proved the existence of horizontal transfer events affecting the symbiotic genes during the coevolution of *R. azibense* with *P. vulgaris* and *V. faba* in their respective distribution centers.

**Fig. 3:**
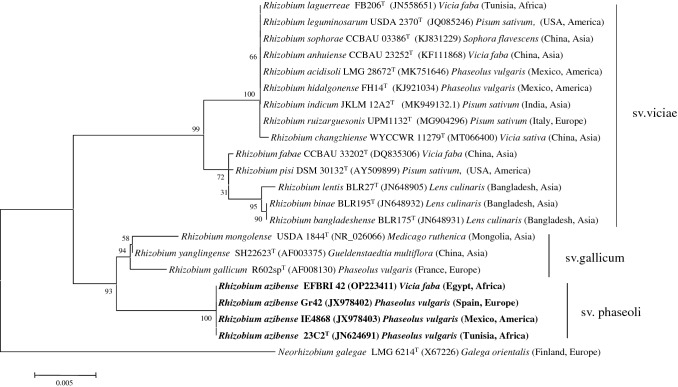
Neighbor-joining phylogenetic tree based on *nodC* gene sequences (370 nt) showing the position of the strain EFBRI 42. Bootstrap values calculated for 1000 replications are indicated. Bar, 2 nt substitution per 100 nt. Accession numbers from Genbank are given in brackets

## Conclusion

In this study, we report for the first time the nodulation of *V. faba* by the species *R. azibense* and the definition of the symbiovar viciae within this species, which to date only contained symbiovars nodulating *P. vulgaris*, such as gallicum and phaseoli. This finding confirmed the existence of horizontal transfer events affecting the symbiotic genes during the coevolution of *R. azibense* with different legume hosts.

## Data Availability

Sequences of 16S rRNA, *recA*, *atpD,* and *nodC* genes of strain EFBRI are deposited in the gene bank under accession numbers OP223411, OP381820, OP381822, and OP381821, respectively.
